# Microscopes of Large Angle of Aperture

**Published:** 1853-01

**Authors:** W. H. Dwinelle


### ARTICLE VII.

*Microscopes of Large Angle of Aperture.* By W. H. DWINELLE, M. D., D. D. S.

SINCE the discovery of Dr. Goring, that the penetrating power of the microscope depends directly upon the angle of aperture of its objectives, all judicious and successful artists have made this discovery the basis of their labors for improvement. All the later advances which have been made, have given additional evidence of the truth of this principle. Indeed, the discovery of Dr. Goring, may be considered as an established law, founded on evidence as complete as any law of physical optics.

In the report of the jury of the great exhibition, on microscopes, and in the expressed opinions of some microscopists of the present day, especially English ones, we have observed an indirect doubt expressed as to the truth of this law.

In conversation a few weeks since, in London, with Mr. John Tomes, one of the best microscopists of England, certainly one of the best in our profession, he distinctly expressed to us, that his experience had led him to regard objectives of great angle of aperture, as of little value for viewing other than the ultimate surfaces of objects, that they were entirely wanting in penetrating power, and that for the examination of objects of structural anatomy, or wherever *depth* is required, an angle of aperture of 75° is superior to all others. This would seem to be the general opinion abroad. Thus the report, and the opinions referred to, agree in the statement, "that it is advisable that the angle of aperture of the combinations, should not be extended to its utmost possible limit, when destined for general purposes of natural history or anatomical investigation. Combinations of extremely extended angle of aperture, are excellent in developing one class of test objects, viz. minute lines, or dots, on plane surfaces, and admirably demonstrate the high perfection to which such glasses are capable of being carried by scientific opticians; but such combinations with a less angle of aperture, and more penetrating power, are far more generally useful to the minute anatomist and naturalist," that is to say, we must *diminish* the angle of aperture, and *increase* the penetrating power, when it is a perfectly established law that penetration is absolutely dependent on angle of aperture! So true is this, that *penetration* and *angle of aperture* are convertible terms.

It may be observed, in passing, that the term *penetration* is an unfortunate one, as every such term must be that admits of doubtful or double meaning. Some have undoubtedly understood by the term, a certain latitude or *depth*, within and without the best focus, by which slight elevations or depressions, below or above the general surface of an object, remain visible and comparatively distinct. It is hardly necessary to say, that this understanding of the term, is an entire mistake.

By penetration, is intended that power by which faint or minute markings are revealed, markings which remain invisible to the most perfect *defining* and *magnifying* powers when accompanied by penetration, a more appropriate term would be *resolving power.*

The existence of latitude, or depth of focus, above referred to, is perfectly incompatible with the highest excellence in an objective, and the presence of such a characteristic, is ample proof of imperfection in the corrections for sphericity. Such objectives cannot give perfect definition, and are invariably blind to the fine markings of objects. The focus of a perfect objective may be fitly called a mathematical plane—extension without depth or thickness; and the more nearly its practical operations coincides with this definition, the more nearly perfect it may be assumed to be.

But to return from this digression. No reasons have been given why the conclusions above named, (against the extension of the angle of aperture,) should be received. So complete a departure from the path in which all past improvements have been made, would seem to have required this. Opinions coming from such a source, always carry a certain weight with them, especially with those who are ignorant of the principles involved, and who must, therefore, take their judgments at second hand. In this instance, the declared conclusions are utterly at variance with both theory and experience. In every instance in which an extension of the angle of aperture has been effected, without the introduction of defects in the corrections for color and figure, the increased efficiency of the microscope has become at once evident and undeniable. We must seek for an explanation of the erroneous conclusions of the jury, in something besides the destruction of an established law, and a denial of the results of all experience.

Fortunately, the explanation is not so difficult as might be supposed. It may be premised that the attempt to increase the angle of aperture of any given objective, implies the necessity of abandoning all, or nearly all, of the curves previously used in its construction, and in many cases, an entire change of material. The addition of a very few degrees to any given angle of aperture, so change the thickness and arrangement of the component lenses of an objective, that the balance of corrections for color and figure is entirely destroyed. It follows from this, that each objective, of given angle, requires an independent investigation of its curves and combinations, to produce the best effect; and it may be added, that after the most searching analytical investigation, there will always remain some practical defects, which can only be corrected by practical means. We can readily understand, therefore, that there is a period in the progress of improvements, during which, imperfect results are likely to be obtained. The practical defects mentioned, can only be remedied after repeated trials, and as the artist cannot afford to bear either the loss of time or the heavy expense involved in such experiments, it follows that his work must leave his hands in an imperfect state during such periods.

The investigations undertaken by Spencer, in this country, and which resulted in the extension of the angle of aperture from 135° to 175°, will serve to illustrate this subject. Although an angle of aperture of 170° was obtained by him at an early day, thus proving that the limit of 135° assigned by Mr. Ross, was erroneous, yet so great a sacrifice of the working focus and of the definition was the result of the earlier formulas, that the artist refused to permit any such objectives to leave his hands. Nearly two years elapsed before these defects were so far overcome as to make such powers practically available.

The results of a recent examination of some of the latest, and ostensibly the best, objectives of Ross, and of Power and Lealand, will probably enlighten us still farther as to the probable grounds of the conclusions arrived at by the jury. The objectives were two of one-fourth of an inch by Ross; and a half inch, a one-fourth, and one-twelfth by Power and Lealand. The two objectives of Ross, differed in their angle of aperture, one of them having the angle usually obtained, in this power, by the maker, was characterized by the usual skill of the artist, in the fine balance of its corrections. The other, of greater angle of aperture, was defective in the corrections for both color and figure, and the difference between the two objectives was so considerable, that the owner, though not able to understand the cause, had early arrived at a correct conclusion respecting their comparative excellence, declaring the one of lesser angle, decidedly the most effective of the two. A careful examination of the deficient objective, revealed the fact, that the artist had endeavored to increase the angle of aperture, mainly by increasing the diameter of the component lenses. Like all such attempts, it was a failure, a result that may safely be predicted in all cases of the kind, however gifted the artist who makes them. The correction for color was not glaringly defective, but the corrections for spherical aberration, were very far from being correctly balanced, a marginal over-correction, and a central under correction, were at once apparent, and of such amount as seriously to impair the definition of the combination.

If such an objective had been brought before the jury of the exhibition, as one of unusually great angle of aperture, and especially as one of Ross' best, (as the owner declared was stated to him,) we can readily imagine what would be the result of the examination. The jury, relying upon the acknowledged skill of Mr. Ross, and believing, with the peculiar faith of Englishmen, that the work of their most distinguished artist, must be perfect of its kind, would most surely have arrived at a judgment adverse to an increase of the angle of aperture in the power under examination, without further warrant for such a judgment, than was supplied by such a faith in the artist and the evident deficiencies of the objective.

The half inch and quarter inch objectives of Power and Lealand, were highly creditable to their makers, the quarter inch being unusually good. They were of the usual angle of aperture of such powers. A careful examination of the one-twelfth, showed that the angle of aperture had been increased at the expense of the definition. It was easily to be seen, also, that the formula used in its construction, was, mainly, a simple reduction of the curves of the quarter inch. The artists had apparently been prompted by the excellence of their one-fourth objectives, to try the application of the formula to the one-twelfth. The result, as above stated, was that the objective, though of larger angle of aperture than usually obtained by the artists, was defective in its corrections for spherical aberrations, or in other words, in its definitions.

If we should assume that these objectives are fair examples of the skill of their respective makers, we might with apparently good reason, arrive at conclusions identical with those of the jury; but in so doing we should be alike unjust to the artists and forgetful of the true principles upon which to form a correct judgment. It does not by any means follow, on the one hand, that the artist must be always unsuccessful in his difficult task, and, on the other, we are not at liberty to cast a shade of doubt on well established laws, because of our inability to separate the deficiencies of the artist from the sound deductions of theory.

Certain it is, that no valid reason can be given against the extension of the angle of aperture of objectives to the greatest possible limit—and for every power—consistent with a due balance of its errors of figure and color throughout the whole area of the objective.

But let us examine briefly into the validity of the conclusions of the jury in another way. The report says, "combinations of extremely extended angle of aperture are excellent in developing one class of test-objects, viz. minute lines or dots on plane surfaces, &c., &c."

From the texture of this sentence, we should be led to suppose that test-objects were things of no general interest, apart from the uses to which they are applied, and that objectives might be made which would give a perfect resolution of the structure of these objects, yet would not possess advantages equal to those of smaller aperture, in the examination of objects of natural history or minute anatomy. Such is a fair translation of the meaning of the sentence. Now, as all of these same test-objects, in general use, are natural objects, whose texture and markings it is of use to the naturalist to make out, and as the same objectives which will enable him to resolve them will enable him to bring out the structure of all of the other objects to the same degree of minuteness, it is evident that the conclusions of the jury are not only erroneous in denying known laws, but are equally false in the assertion of principles that have no existence. Indeed, on the next page of the report, the jury in making mention of the objectives of Smith and Beck, says, "the half-inch focus of 70° aperture is a wonderfully fine combination, easily showing objects considered difficult, for one-eighth inch focal length, a little more than a year since, and bearing the application of the higher eye-pieces in an unprecedented manner." Now it might be fairly asked of the jury to explain upon what peculiarity the excellence of this half-inch objective (the one of largest angle of aperture, for this power, mentioned in the report) depended. It is perfectly notorious, so far as mere definition is concerned, that the best artists of the present day have not advanced one step beyond the triple achromatics of Tulley, made in 1823. It is equally true that the discoveries of Lister, while they have enabled artists to obtain larger angles of aperture, with due balance of the errors of color and figure, have added nothing to the defining power of objectives.

But a still more searching objection to the conclusions of the jury must be made; and one, too, drawn from the very departments of research to which they have made especial reference. As above stated, the focus of a perfect objective is, as nearly as may be, a mathematical plane, and, such being the case, all objects of whatever kind, shape, size or character, are examined under nearly the same circumstances—the structure only of such objects, or parts of objects as are accurately in focus, can be examined with any certainty. Nearly all of the objects and structures which are made the subjects of study or investigation by the microscopic anatomist and naturalist, are transparent ones, and are examined by transmitted light. In order to rid the examination of these objects of the confusion of varying refractions, diffractions and interference, it is necessary that nearly all of them should be mounted in fluid or balsam. The advantages thus obtained are sufficiently great to compensate for the defects belonging to this method of examination. One of its defects, however, is frequently felt as a serious inconvenience. The refractive power of the fluids used is often so nearly that of the structures themselves, that, with objectives of feeble penetrating power, they may disappear altogether, or at most exhibit a bare meagre outline. For this defect there is but one remedy—that of using objectives of large angle of aperture which alone possess the capacity to take up pencils of high incidence and under which the slight difference in refractive power become most apparent. As by far the greater share of all microscopic objects are of the character above indicated, the superiority of objectives of the largest attainable angle of aperture, is so evident alike from the sound deductions of theory and the indisputable test of all past experience, that the only wonder is that any doubt should have been entertained on the subject.

We will, therefore, close these lengthy strictures, with the expression of the hope, that hereafter the deficiencies of the microscope may be traced to their true cause—that of deficient skill in the artists who furnish them.

